# Association of Preoperative Serum Carcinoembryonic Antigen and Gastric Cancer Recurrence: A Large Cohort Study

**DOI:** 10.7150/jca.47899

**Published:** 2021-01-01

**Authors:** Qinbo Cai, Wen Zhou, Jin Li, Xinde Ou, Chuangqi Chen, Shirong Cai, Weiling He, Jianbo Xu, Yulong He

**Affiliations:** 1 Center of Gastrointestinal Surgery, the First Affiliated Hospital of Sun Yat-sen University, Guangzhou, P. R. China.; 2 Center for Diagnosis and Treatment of Gastric Cancer, Sun Yat-sen University, Guangzhou, P. R. China.; 3 Laboratory of General Surgery, the First Affiliated Hospital of Sun Yat-sen University, Guangzhou, P. R. China.; 4 Center for Digestive Disease, the Seventh Affiliated Hospital of Sun Yat-sen University, Shenzhen, P. R. China.

**Keywords:** Gastric cancer, CEA, recurrence, prognostic factor

## Abstract

**Background and Aim:** Measuring postoperative carcinoembryonic antigen (CEA) is recommended by guidelines to help detecting recurrence of gastric cancer patients. However, the prognostic significance of elevated preoperative CEA is unclear. This study aims to investigate whether patients with elevated preoperative CEA have a higher risk of recurrence than patients with normal preoperative CEA.

**Methods:** We conducted a retrospective cohort study at a gastric cancer center in South China. Consecutive patients with stage I to III gastric adenocarcinoma who underwent curative resection at the center from January 2001 to February 2016 were identified. Patients were grouped into two cohorts: normal preoperative CEA (≤ 5 ng/ml), and elevated preoperative CEA (> 5 ng/ml). 3-year recurrence-free survival (RFS) and hazard function curves over time were estimated.

**Results:** A total of 1,596 patients (1,063 {66.6%} male; median {Interquartile range, IQR} age, 59 {50-66} years) were identified. Patients with elevated preoperative CEA had 15.5% lower 3-year RFS (n=222 {70.4%}) than the cohorts with normal preoperative CEA (n=1,374 {85.9%}). The hazard function of recurrence for the two cohorts peaked at the similar time (around 10 months after surgery). Multivariate Cox analyses confirmed that elevated preoperative CEA was independently associated with shorter RFS (Hazard Ratio {HR}, 1.69; 95% confidence interval {CI}, 1.26-2.27; *P* = 0.001).

**Conclusions:** Patients with elevated preoperative CEA are at increased risk for recurrence, especially within the first 24 months after surgery.

## Introduction

Carcinoembryonic antigen (CEA) is recommended by national guidelines as a tumor marker in gastric cancer 1, 2. Previous studies had controversial results about association between preoperative serum CEA and overall survival of gastric cancer even in large sample size cohorts 3-7. Due to lack of high quality evidence, level of preoperative CEA is not considered as independent prognostic factor for gastric cancer, and cure strategy should not be changed based on preoperative CEA level according to the 8^th^ edition of American Joint Committee (AJCC) staging system 8. Overall survival is affected by various non-cancer factors, such as non-gastric cancer diseases and income, which are hard to be adjusted in multivariate analysis and might lead to bias. Recurrence heralds a worse prognosis after radical resection of gastric cancer 9 and is supposed to be an indicator for cancer-specific outcome. Measuring postoperative CEA has been recommended by guidelines to help detecting recurrence 1, 2, 8, 10, but the role of preoperative CEA in predicting recurrence is still unclear. There is not cohort with large size of patients reporting the association between preoperative CEA and recurrence of gastric cancer. In this study, we aimed to determine whether preoperative serum CEA is a prognostic factor for recurrence of gastric cancer after radical resection in a large sample size cohort.

## Materials and Methods

### Study design and patient cohort

**Figure 1:**
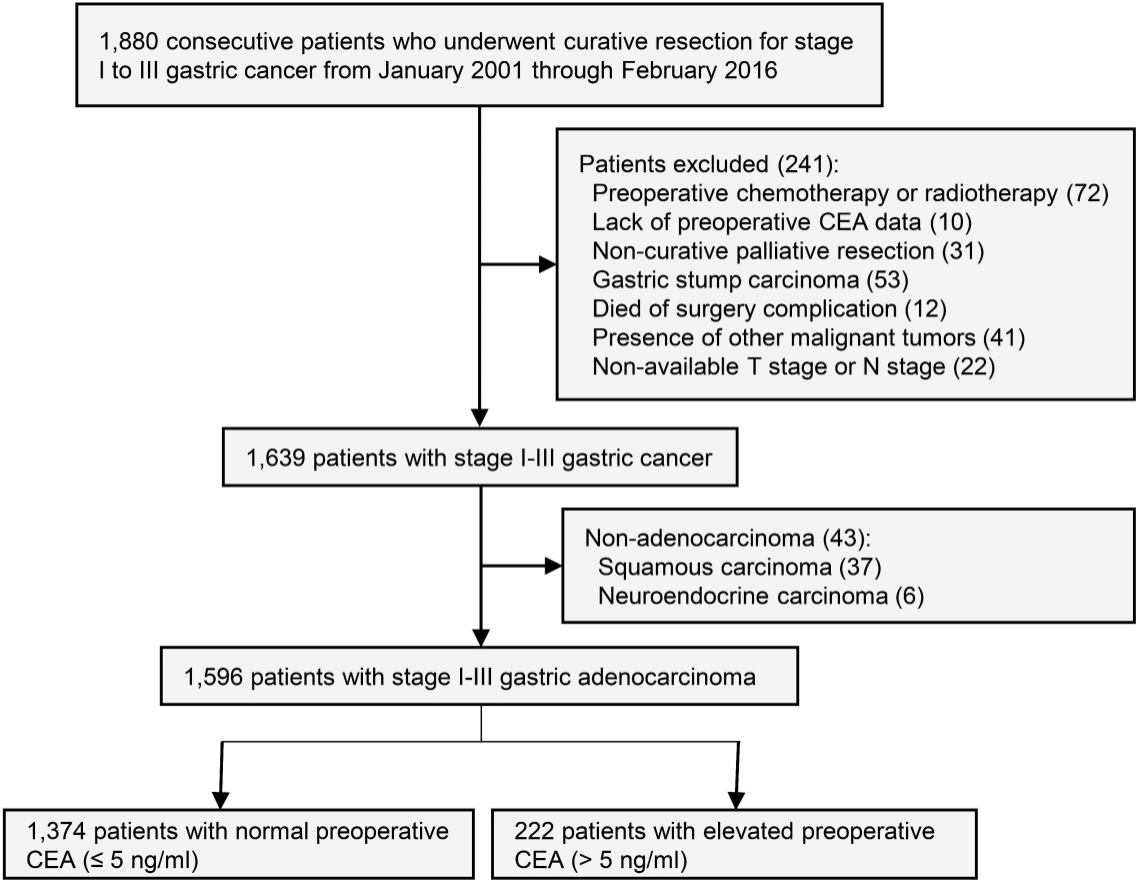
Study design.

This study was approved by the institutional review board of the First Affiliated Hospital of Sun Yat-sen University. Informed consent of study patients was waived by the review board. Inclusion criteria: Prospectively maintained databases were queried for all consecutive patients who underwent curative surgery for stage I to III gastric cancer patients from January 2001 to February 2016 at gastric cancer center of the First Affiliated Hospital of Sun Yat-sen University. The last follow-up date of this study was February 2018. Exclusion criteria: Preoperative chemotherapy or radiotherapy, lack of preoperative CEA data, non-curative palliative resection, gastric stump carcinoma, death of surgery complication, presence of other malignant tumors, non-available T stage or N stage, and non-adenocarcinoma (**Figure 1**).

Preoperative serum CEA was defined as the CEA value closest to surgical date. Patients were grouped as follows: (1) Normal preoperative CEA group (≤ 5 ng/ml); (2) Elevated preoperative CEA group (> 5 ng/ml). The CEA value was measured at the First Affiliated Hospital of Sun Yat-sen University using an Abott ARCHITECT analyzer (Abott, USA). The reference normal range was 0.0 to 5.0 ng/ml.

### Staging and follow-up

The preoperative stage was determined by contrast-enhanced computed tomography (CT) of the chest, abdomen and pelvis. The original pathologic TNM stages of patients in the databases were based on the 6^th^ and 7^th^ AJCC on Cancer staging system. The original pathological reports were reviewed by clinician, and then modified all the pathologic stages according to the 8^th^ edition 8. Adjuvant chemotherapy was administered to patients after histological evaluation of the surgical specimen according to the National Comprehensive Cancer Network (NCCN) guidelines. Postoperative follow-up was performed every 3-6 months for the first 3 years, then every 12 months from years 4 to 5. The routine patient follow-up appointments included a physical examination, laboratory tests, chest radiography, abdominal ultrasonography, CT or positron emission computed tomography (PETCT) and an annual endoscopic examination. CT was the most frequently used imaging method, including chest, abdomen and pelvis with intravenous contrast. Diagnosis of recurrence was based on new lesions on CT, PETCT or histological confirmation through biopsy.

### Statistical analysis

Continuous variables were compared using the Mann-Whitney U test. Categorical variables were compared using the Chi-square test or Fisher's exact test. The recurrence-free survival (RFS) period was defined as the period from the date of surgery to the date of recurrence or last follow-up without recurrence. Patients who died without known tumor recurrence were censored at the last documented evaluation 11. Differences in RFS were assessed by the Log-rank test. Hazard ratio (HR) and 95% confidence interval (CI) were estimated using Cox regression models and evaluated by the Wald test. Variables with* P* value less than 0.05 on univariate analyses were included in the multivariate analysis. The hazard function of recurrence was estimated using Kernel-based method 12, 13. All statistical analyses were performed using R version 3.6.1 (R project) and IBM SPSS software (version 22, New York, USA). All tests were two-sided and *P* values of less than 0.05 were considered significant.

### The Cancer Genome Atlas (TCGA) stomach adenocarcinoma analysis

We downloaded mRNA expression data and clinical information of the TCGA stomach adenocarcinoma program from cBioPortal. Patients with stage I-III gastric adenocarcinoma were included. Exclusion criteria as follows: Patients with preoperative chemotherapy or radiotherapy, or with other malignant tumor. Patients with CEA cell adhesion molecule 5 (*CEACAM5,* name of gene encoding protein CEA) expression in the top 20% range were divided into high expression group, and the rest 80% were divided into low expression group.

### *In silico* mechanism analysis

We used cBioPortal to identify genes correlated with *CEACAM5* in mRNA expression level. Genes with an adjusted-*P* value less than 0.01 were considered significantly correlated with *CEACAM5* and used for Gene Oncology (GO) enrichment and Kyoto Encyclopedia of Genes and Genomes (KEGG) pathways analyses. The clusterProfiler R package was used for analysis.

## Results

A total of 1,596 patients (1,063 {66.6%} male; median {Interquartile range, IQR} age, 59 {50-66} years) were identified. Characteristics of the 1,596 patients with normal or elevated preoperative CEA were shown in **Table 1.** In this study, 1,374 patients were grouped into the normal preoperative CEA cohort with a median (IQR) CEA level of 1.6 (0.99-2.49) ng/ml. The elevated preoperative CEA cohort included 222 patients and the median (IQR) CEA level was 11.09 (7.04-27.18). The median (IQR) follow-up time for all patients was 37.68 (18.17-60.00) months. A total of 253 patients (15.9%) had recurrence before the last follow-up. The 3-year RFS rate for all patients was 83.8% (95% CI, 81.8%-85.9%).

**Figure 2:**
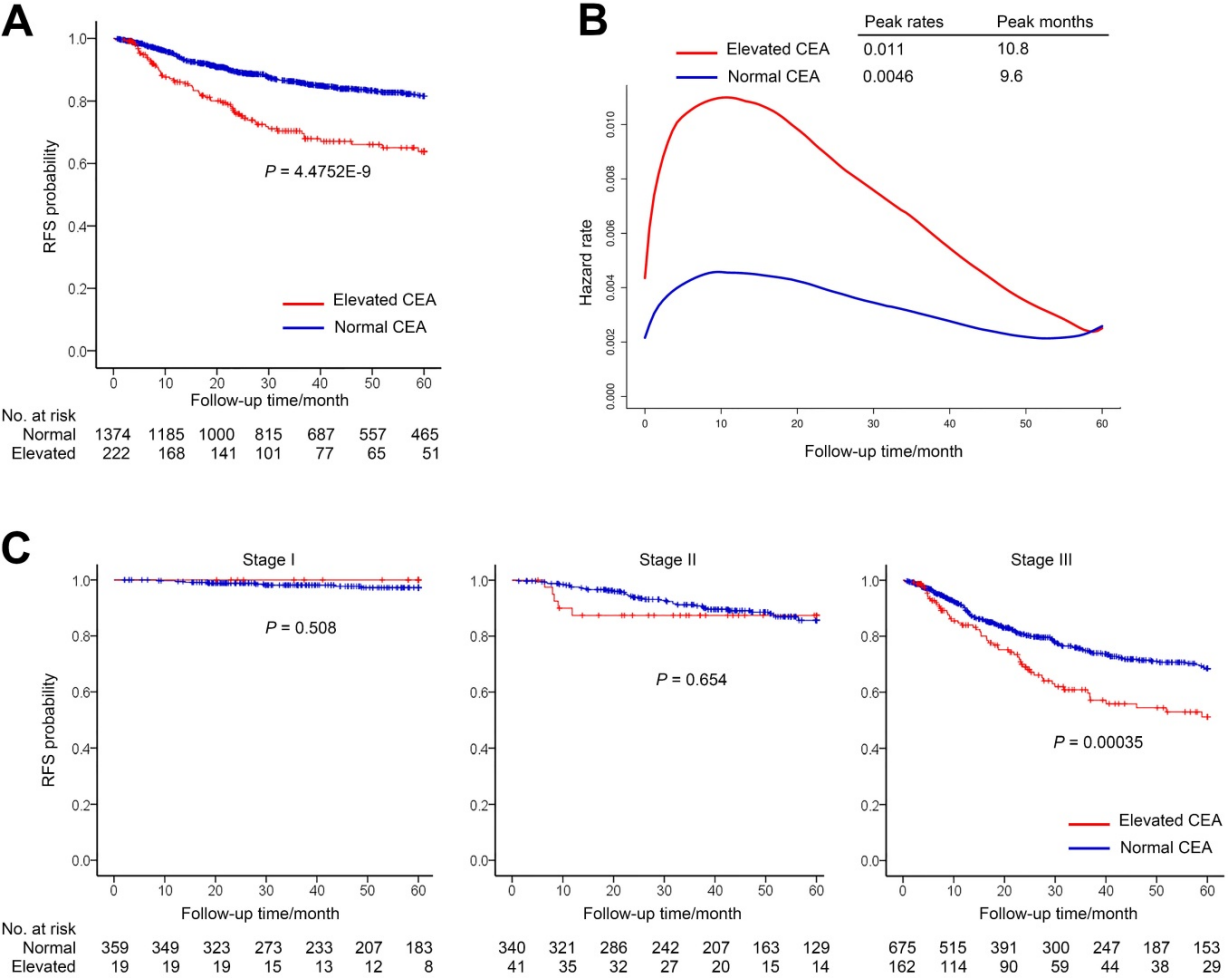
Recurrence-free survival (RFS) by preoperative CEA. (A) RFS of patients with normal preoperative CEA vs elevated preoperative CEA. (B) Hazard functions for the disease recurrence in the two cohorts. (C) Stage-specific analyses of RFS based on preoperative CEA level.

In the normal group and the elevated group, the median (IQR) follow-up periods were 39.92 (18.9-60) and 25.69 (10.69-58.02) months, respectively. The 1-year RFS rate for patients with elevated preoperative CEA was 86.1% (95% CI, 81.4%-91.0%) compared with 94.8% (95% CI, 93.6%-96.0%) for patients with normal preoperative CEA. The 3-year RFS rate for elevated group was 70.4% (95% CI, 63.9%-77.5%) compared with 85.9% (95% CI, 83.9%-88.0%) for normal group (**Figure 2A**).

The smooth curve of the hazard function indicated that the risk of recurrence was higher in the elevated preoperative CEA group (**Figure 2B**). Nevertheless, both of the two groups peaked at the similar time and had high recurrence risk during the first 2 years after surgery.

We further investigated relationship between RFS and CEA in different stage patients (**Figure 2C**). The RFS of two cohorts had no significant difference in patients with stage I or II gastric cancer (**Figure 2C**). However, among patients with stage III gastric cancer, RFS was significantly lower in the elevated preoperative CEA cohort than that of normal preoperative CEA cohort (3-year RFS rate, 60.9% vs 74.8%, Log-rank *P* = 3.5E-04) (**Figure 2C**).

Univariate and multivariate analyses for risk factors associated with RFS are shown in **Table 2.** In univariate analyses, tumor located in entire stomach, poor differentiation, higher TNM stage, no adjuvant chemotherapy and elevated preoperative CEA were associated with shorter RFS. Multivariate analysis indicated that elevated preoperative CEA was independently associated with shorter RFS (HR = 1.69, 95% CI = 1.26-2.27, *P* = 0.001) together with tumor located in entire stomach, poor differentiation and higher TNM stage.

The recurrence patterns of the two cohorts were shown in **Table 3.** It was interesting that patients with elevated preoperative CEA was more likely to have liver metastasis rather than peritoneal metastasis.

To validate whether the mRNA level of CEA was associated with recurrence of gastric cancer, we downloaded mRNA expression data of *CEACAM5* and clinical information from cBioPortal. Results showed that high expression of *CEACAM5* was significantly associated with poor RFS. The 3-year RFS rate for high-expression group was 48.3% (95% CI, 34.4%-68.0%) compared with 58.1% (95% CI, 50.5%-66.8%) for low-expression group (Figure S1A). The same trend was observed in the subgroup analyses of each stage. However, there was no significant statistical difference, which might be due to the small sample size in each stage (Figure S1 B-D).

In order to investigate the possible mechanisms associated with CEA and gastric cancer recurrence, cBioPortal was used to analyze genes correlated with *CEACAM5* in mRNA expression level. Finally, 3,286 genes with an adjusted-*P* value less than 0.01 were considered significantly correlated with *CEACAM5* and included for GO enrichment and KEGG signaling pathways analyses (Table S1). As a membrane protein, CEA was associated with cell-cell adhesion and junction (Figure S2) 14. Surprisingly, as indicated by results from both GO and KEGG, CEA was significantly associated with chemokine signaling and immunology regulation, especially T cells and Th cells (Figure S2), which were already known to mediate cancer metastasis in previous studies 15-17.

## Discussion

In this study, we observed that patients with elevated preoperative CEA have a 15.5% lower 3-year RFS than those with normal preoperative CEA. The hazard function curve further demonstrated the impact of elevated CEA and shown a higher peak in the elevated CEA cohort compared with the normal CEA cohort. Multivariate Cox regression also confirmed that elevated CEA was an independent prognostic factor for recurrence of gastric cancer. In stratified analysis, we observed that preoperative CEA could stratify patients with stage III rather than those with stage I or II, though patients with elevated CEA have a 10.4% lower 1-year RFS than those with normal CEA in stage II without statistical significance. This difference is likely due to the limited recurrence in stage I group, which has a 3-year RFS greater than 98%.

Preoperative CEA was reported as an independent prognostic factor for RFS in a retrospective cohort of 621 patients by multivariate Cox model, adjusted by for age, stage, NUAK family kinase 2 (NUAK2), pyruvate dehydrogenase kinase 1 (PDK1), phospho-AMP-activated protein kinase (pAMPK) and mitogen activated kinase (MAPK) 3/1 18. Other studies also reported elevated preoperative CEA predicted shorter RFS under unadjusted condition 19-21, while some studies showed negative results without adjustment 22, 23, and the sample size of these studies vary from 70 to 479. The results from these studies are inconsistent, which might be due to the small sample size and unadjusted analyses. In this study, we demonstrated the prognostic value of preoperative CEA using multivariate analysis in a large cohort of 1,596 consecutive patients. We intend to provide some evidence for clinic practice.

The important role of postoperative CEA in cancer surveillance has been widely accepted 1, 2, 8, 10. Since some elevated preoperative CEA normalized after surgery 5, people may argue that preoperative CEA is not needed for follow-up. However, recurrence can be accompanied by normal or elevated postoperative CEA 22. The value of elevated preoperative CEA is to define a small proportion of patients (13.9% in this study, 16.6% to 28.8% in other studies 3, 5, 6) with higher risk of recurrence compared to patients with normal preoperative CEA in the same stage. These patients are supposed to undergo more intensive surveillance plan. Besides serum CEA, positive of preoperative CEA mRNA in peritoneal lavages predicts the peritoneal recurrence 24, which confirms the role of CEA in defining patients with high risk of recurrence.

This study indicates that patients with elevated CEA were more likely to have liver metastasis than those with normal CEA as previously reported 25, 26. Higher CEA is also associated with positive vessel carcinoma embolus 27. It seems that tumors with elevated CEA are more invasive and prefer hematogenous metastasis. CEA is shown to inhibit transforming growth factor-β (TGF-β) signaling 28 and promotes liver metastasis of colon cancer in mice model 28, 29.

The major strengths of this study include large size of the cohort, patients with high-quality preoperative CT and standardized pathological reports; the uniform treatment patients received, with standard D2 or D2+ resection technique by specialized gastric surgeons and retrieved lymph node more than 16 in 83.2% of patients in this cohort; and standard adjuvant chemotherapy in all patients. Standard chemotherapy was handled by a special group in our center over years. This cohort has similar oncological outcomes to those seen in other reports from centers in Japan and Korea, where standard D2 resection technique is commonly performed. For example, the recurrence rate in this cohort is similar as previous report 30.

## Limitations

This study is with limitation inherent in observational retrospective cohorts. First, intervals and completeness of the follow-up were varied, although postoperative follow-up was performed according to national guideline 1. Second, patients who died without known tumor recurrence were censored in this study. It is possible that some of them died because of recurrence without imaging test, which leads to underestimation of recurrence rate and potential bias. In addition, we did not control known factors that affect CEA, such as tobacco use, nonmalignant gastrointestinal disorders, lung disease, and hypothyroidism 31, 32. Furthermore, this study is implemented in a single center and it would have some limitations to apply to other centers. Further validation studies are warranted to explore the value of preoperative CEA as an independent prognostic factor.

## Conclusion

The elevated preoperative CEA independently predicts shorter RFS for patients with radically resected gastric cancer. Patients with elevated CEA tend to be under significant higher risk of recurrence in the first 2 years after surgery, which might provide evidence for risk-adjusted individualized surveillance strategy.

## Supplementary Material

Supplementary figures.

Supplementary table S1.

## Figures and Tables

**Table 1:** Patients and tumor characteristics

Characteristics	CEA ≤ 5 ng/ml (n=1,374)	CEA > 5 ng/ml (n=222)	*P*
**Gender, n (%)**			**< 0.001**
Male	889 (64.7)	174 (78.4)	
Female	485 (35.3)	48 (21.6)	
**Age, years, median (IQR)**	58 (49-66)	63 (55-69)	**< 0.001**
**Primary site, n (%)**			**< 0.001**
Upper third	354 (25.8)	93 (41.9)	
Middle third	358 (26.1)	51 (23.0)	
Lower third	620 (45.1)	72 (32.4)	
Entire	41 (3.0)	6 (2.7)	
Unknown	1 (0.1)	0 (0)	
**Tumor differentiation, n (%)**			0.862
G1/2	355 (25.8)	61 (27.5)	
G3/4	990 (72.1)	156 (70.3)	
Gx	29 (2.1)	5 (2.3)	
**No. of retrieved lymph nodes, Median (IQR)**	29 (12-41)	32.5 (21-42.25)	**0.036**
**Pathologic T stage, n (%)**			**< 0.001**
Tis/T1	280 (20.4)	14 (6.3)	
T2	146 (10.6)	16 (7.2)	
T3	188 (13.7)	32 (14.4)	
T4a	666 (48.5)	136 (61.3)	
T4b	94 (6.8)	24 (10.8)	
**Pathologic N stage, n (%)**			**< 0.001**
N0	601 (43.7)	47 (21.2)	
N1	244 (17.8)	43 (19.4)	
N2	258 (18.8)	50 (22.5)	
N3a	180 (13.1)	51 (23.0)	
N3b	91 (6.6)	31 (14.0)	
**AJCC 8^th^ ed. pathologic stage, n (%)**			**< 0.001**
I*	359 (26.1)	19 (8.6)	
II	340 (24.7)	41 (18.5)	
III	675 (49.1)	162 (73.0)	
**Adjuvant chemotherapy, n (%)**			0.170
Yes	816 (59.4)	121 (54.5)	
No	558 (40.6)	101 (45.5)	

*There were 19 patients with stage 0 disease and they were grouped into stage I during analyses.

**Table 2:** Univariate and multivariate analyses of recurrence-free survival

Characteristics	Univariate analysis			Multivariate analysis		
	HR	95% CI	*P*	HR	95% CI	*P*
**Gender**						
Male	Ref					
Female	1.17	0.90-1.51	0.236			
**Age, years**						
≤ 55	Ref					
55-60	0.79	0.53-1.16	0.226			
60-65	1.05	0.73-1.50	0.788			
> 65	1.14	0.85-1.54	0.387			
**Primary site**						
Upper third	0.50	0.28-0.89	**0.019**	0.69	0.38-1.25	0.224
Middle third	0.42	0.24-0.77	**0.005**	0.68	0.38-1.24	0.208
Lower third	0.30	0.17-0.54	**< 0.001**	0.54	0.30-0.97	**0.039**
Entire	Ref			Ref		
**Tumor differentiation**						
G1/2	Ref			Ref		
G3/4	2.08	1.49-2.90	**< 0.001**	1.65	1.17-2.33	**0.004**
**No. of retrieved lymph nodes**						
< 16	Ref					
≥ 16	1.24	0.88-1.75	0.224			
**AJCC 8^th^ ed. pathologic stage**						
I	Ref			Ref		
II	5.63	2.64-11.98	**< 0.001**	5.21	2.32-11.69	**< 0.001**
III	17.13	8.45-34.73	**< 0.001**	14.71	6.83-31.67	**< 0.001**
**Adjuvant chemotherapy**						
Yes	Ref			Ref		
No	1.67	1.30-2.14	**< 0.001**	1.01	0.79-1.31	0.923
**CEA**						
≤ 5 ng/ml	Ref			Ref		
> 5 ng/ml	2.31	1.73-3.09	**< 0.001**	1.69	1.26-2.27	**0.001**

**Table 3:** Analysis of sites of recurrence in relation to CEA status

Recurrence site	CEA ≤ 5 ng/ml, n (%)	CEA > 5 ng/ml, n (%)	*P*^#^
***Total sites***	185	55	
**Local**			0.059
Present	25 (13.51)	14 (25.45)	
Absent	160 (86.49)	41 (74.55)	
**Peritoneal**			**0.003**
Present	78 (42.16)	11 (20.00)	
Absent	107 (57.84)	44 (80.00)	
**Lymph node**			0.372
Present	28 (15.14)	5 (9.09)	
Absent	157 (84.86)	50 (90.91)	
**Liver**			**0.011**
Present	29 (15.68)	18 (32.73)	
Absent	156 (84.32)	37 (67.27)	
**Lung**			0.822
Present	24 (12.97)	8 (14.55)	
Absent	161 (87.03)	47 (85.45)	
Other sites*			0.414
Present	14 (7.57)	6 (10.91)	
Absent	171 (92.43)	49 (89.09)	

*Including bone, brain, pancreas, adrenal gland and kidney. ^#^Fisher's exact test.

## Abbreviations

AJCC: American Joint Committee
CEA: carcinoembryonic antigen
*CEACAM5*: CEA cell adhesion molecule 5
CI: confidence interval
CT: computed tomography
GO: gene oncology
IQR: interquartile range
KEGG: Kyoto Encyclopedia of Genes and Genomes
MAPK: mitogen activated kinase
NCCN: National Comprehensive Cancer Network
NUAK2: NUAK family kinase 2
pAMPK: phospho-AMP-activated protein kinase
PETCT: positron emission computed tomography
PDK1: pyruvate dehydrogenase kinase 1
RFS: recurrence-free survival
TCGA: The Cancer Genome Atlas
TGF-β: transforming growth factor-β
