# Supplementary material for: Association of Preoperative Serum Carcinoembryonic Antigen and Gastric Cancer Recurrence: A Large Cohort Study
Source: J Cancer. 2021 Jan 1;12(2):397–403. doi: 10.7150/jca.47899 (PMC7738990; doi:10.7150/jca.47899)
Supplement: Supplementary file 1 — Supplementary figures. [file jcav12p0397s1.pdf]

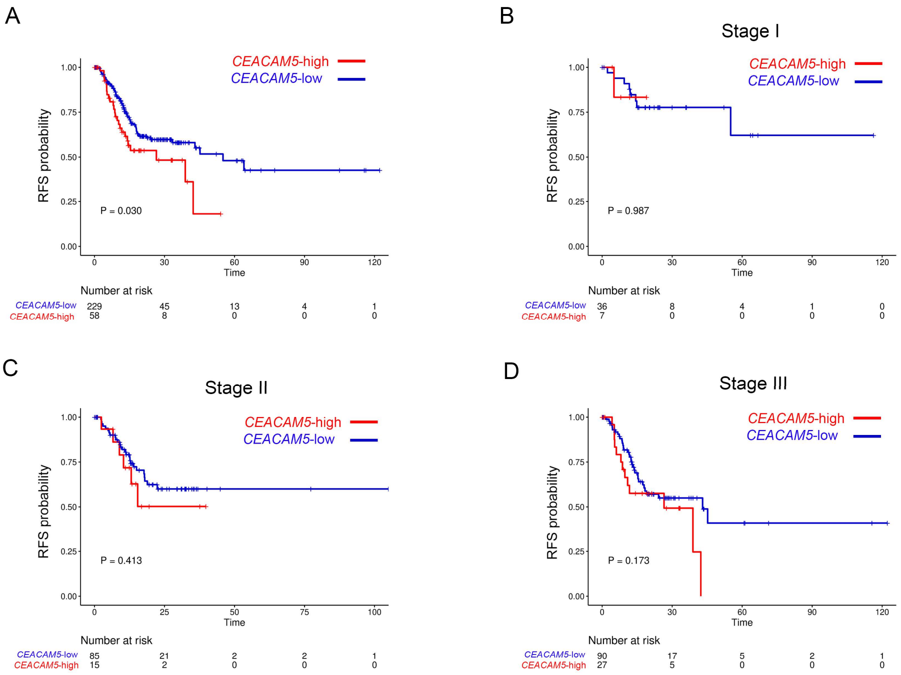

Figure S1 Analysis of association between *CEACAM5* mRNA level and recurrence-free survival (RFS) in TCGA.

(A) RFS in all stage I-III patients. (B-D) RFS in separate TNM stages. A top 20% cut-off of *CEACAM5* mRNA level was used to divide patients into *CEACAM5* high and low groups.

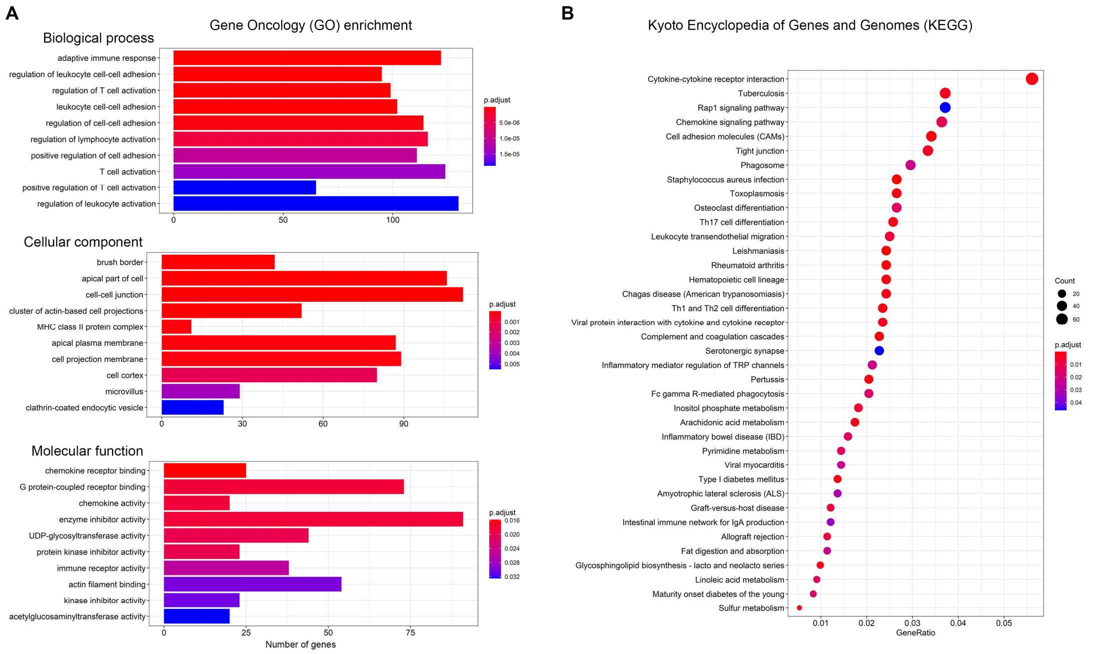

Figure S2 In silico analyses of possible mechanisms associated with CEA.

(A) Gene Oncology (GO) enrichment analysis. Top 10 of each category were shown. (B) Kyoto Encyclopedia of Genes and Genomes (KEGG) pathways analyses. Pathways with an adjusted-*P* value less than 0.05 were shown.
